# Data on heavy metal and magnetic relationships in coastal sediments from South East Coast of Tamilnadu, India

**DOI:** 10.1016/j.dib.2017.11.056

**Published:** 2017-11-20

**Authors:** R. Ravisankar, N. Harikrishnan, A. Chandrasekaran, M. Suresh Gandhi, R. Alagarsamy

**Affiliations:** a Post Graduate and Research Department of Physics, Government Arts College, Tiruvannamalai 606603, Tamil Nadu, India; b Department of Physics, SSN college of Engineering, Chennai 603110, Tamil Nadu, India; c Department of Geology, University of Madras, Guindy Campus, Chennai 600025, Tamil Nadu, India; d National Institute of Oceanography, Council of Scientific and Industrial Research (CSIR), Donapaula, Goa 403004, India

**Keywords:** Sediment, Heavy metals, Magnetic measurements, Pollution

## Abstract

In this data, the heavy metal concentration and magnetic susceptibility in coastal sediment samples collected from Periyakalapet to Parangaipettai of East Coast of Tamilnadu using Energy Dispersive X-ray Fluorescence (EDXRF) technique and dual frequency susceptibility meter. We aimed to (i) determine the heavy metal concentration in the sediments from Periyakalapet to Parangaipettai of East Coast of Tamilnadu (ii) assess the magnetic mineral property of sediments (iii) study the correlation between heavy metal and magnetic susceptibility. The determined heavy metal concentration found in the order of Mn> Ba > V > Cr > Zn > La > Ni >Pb> Co > As > Cd > Cu > Al > Fe >Ca> Ti > K > Mg. The magnetic susceptibility (χ_lf_) measurements show that they vary from 5.92×10^−8^ m^3^ kg^−1^to 29.06×10^−8^ m^3^ kg^−1^ with an average of 20.39×10^−8^ m^3^ kg^−1^. Analysed data confirmed that magnetic susceptibility has the potential tool to indicate the heavy metal pollution sources.

**Specifications Table**

**Table:** 

Subject area	Physics
More specific subject area	Heavy metal and magnetic measurements
Type of data	Table
How data was acquired	Energy Dispersive X-ray Fluorescence Spectrometer (EDXRF) and Dual frequency susceptibility meter.
Data format	Raw data
Experimental factors	The sediment samples were oven dried at 105 °C for 2 h to become a constant weight and grounded into a fine powder using an agate mortar and pestle. Then powder samples were sieved using a <63 μm sieve and stored in desiccators to remove traces of water from the sample until they were analyzed. One gram of the fine grinded sample and 0.5 g of boric acid (H_3_BO_3_) was mixed. The mixture was thoroughly grinded and pressed to a pellet of 25 mm diameter using a hydraulic press (20 t) for EDXRF analysis.
	The dried samples were then sieved using a 1 mm sieve mesh to remove particles such as glass, plant debris, refuse and small stones. The sieved samples were stored in a plastic container for further laboratory measurements. The magnetic susceptibility measurements were then carried out on the sieved samples packaged in a 10 ml plastic container at laboratory temperature. Measurements of magnetic susceptibility were made at both low (0.465 kHz) and high (4.65 kHz) frequencies using MS2B dual frequency susceptibility meter linked to a computer operated using a Multisus2 software.
Experimental features	Determination of concentration of Mg, Al, K, Ca, Ti, Fe, V, Cr, Mn, Co, Ni, Zn, As, Cd, Ba, La, Pb using EDXRF.
	Measurement of magnetic susceptibility were made at both low (0.465 kHz) and high (4.65 kHz) frequencies using MS2B dual frequency susceptibility meter. The percent-frequency dependent susceptibility (χ_fd_%) is calculated using the standard formula.
Data source location	Periyakalapet to Parangaipettai, East Coast of Tamilnadu, India
Data accessibility	Data is with this article.

**Value of the data**

• Data presented on the heavy metals concentration, in sediments can be useful to draw a base line data in marine environment.
• Data shown here used as a tool for anthropogenic causes in heavy metal content and to identify common pollution sources.
• Data shows that continuous monitoring and efforts of remediation might be required to improve the coastal environment near industrialized areas.

## 1 Data

**Fig. 1:**
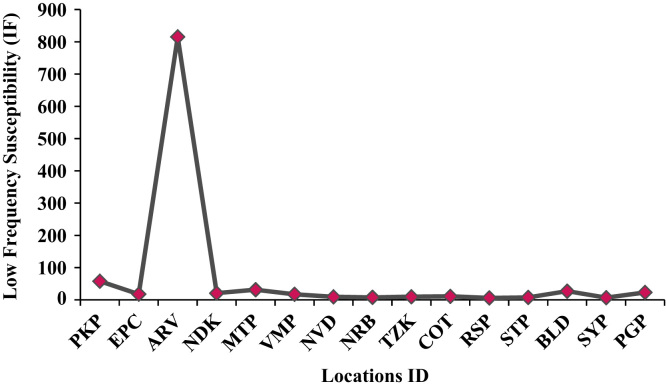
Locatons Vs Low Frequency Susceptibility (IF).

**Fig. 2:**
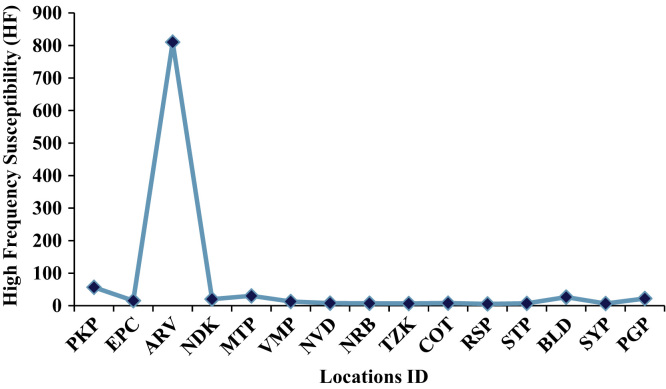
Locatons Vs High Frequency Susceptibility (HF).

The concentration of elements in sediments from Periyakalapet to Parangipettai along the East Coast of Tamilnadu is presented in Table 1. The concentration (mg/kg) varies from 20 to 4200; 15800–27900; 7200–9500; 5800–12500; 376–9889; 3215–21836; 22.7–162.2; 19.2–61.9; 61.4 – 386.9; 1.1–7.1; 16.3–25.8; 22.7 -121.9; 4.7–8.4; 0.2–13.7; 302.9–485.2; 1.1-123 and 2.3 -25.8 for Mg, Al, K, Ca, Ti, Fe, V, Cr, Mn, Co, Ni, Zn, As, Cd, Ba, La, Pb respectively. The mean concentration values of heavy metals in sediments do not exceed the natural background levels of heavy metals given by Turekian and Wedepohl, (1961) [1]. This indicates that study area dominated with large amount of natural sediment with low heavy metal content [2]. Table 2 shows Low Frequency Susceptibility (IF) (10^−8^ m^3^/kg), High Frequency Susceptibility (HF) (10^−8^ m^3^/kg) and Frequency dependent susceptibility (FD) % and Fig. 1, Fig. 2 shows the location Vs Low Frequency (χ_lf_) and high frequency Susceptibility (χ_hf_).

**Table 1:** Heavy metal concentration of the sediment samples along the study area.

**S. No**	**Element**	**Mg**	**Al**	**K**	**Ca**	**Ti**	**Fe**	**V**	**Cr**	**Mn**	**Co**	**Ni**	**Cu**	**Zn**	**Cd**	**Ba**	**La**	**Pb**
		ppm	ppm	ppm	ppm	ppm	ppm	ppm	ppm	ppm	ppm	ppm	ppm	ppm	ppm	ppm	ppm	ppm
1	PKP	2223	20696	6615	8943	2039	9534	50.11	42.38	192.26	3.38	20.86	BDL	30.54	5.5	312.4	12.9	4.4
2	EPC	25	20255	6202	7239	2340	8458	50.9	30.3	180.1	2.8	19.8	BDL	23.0	2.1	306.1	29.1	1.5
3	ARV	1800	37425	5484	8070	51434	57902	711.0	207.3	1387.6	19.0	24.4	BDL	89.0	BDL	180.2	216.7	35.7
4	NDK	300	13532	6800	4592	530	3647	23.4	12.5	68.1	1.1	15.2	BDL	14.0	BDL	411.9	BDL	BDL
5	MTP	1028	19066	7869	7406	1216	5520	26.37	21.21	110.05	1.88	16.48	BDL	20.16	10.2	385.4	BDL	1.4
6	VMP	6007	30893	5044	20809	15464	35269	234.71	127.00	750.16	12.51	33.63	BDL	62.31	3.4	209.0	47.0	17.0
7	NVD	3022	26895	4468	21176	11689	33771	204.56	123.33	748.38	11.95	33.30	BDL	65.67	BDL	152.3	31.0	19.8
8	NRB	5051	31132	4850	21679	19539	40489	310.87	155.77	869.09	14.35	30.23	3.60	65.94	BDL	176.0	51.2	25.5
9	TZK	816	21212	6085	12057	3357	13407	64.94	54.52	243.11	5.01	23.21	BDL	30.78	1.4	256.7	19.1	9.1
10	COT	1608	19866	5392	11628	3776	13137	71.38	55.33	263.74	4.61	24.59	BDL	29.00	3.6	236.1	6.4	6.1
11	RSP	795	23554	7286	11363	931	8308	31.85	43.85	157.81	3.10	22.84	BDL	22.47	2.3	308.2	BDL	6.8
12	STP	1773	22928	9350	11586	724	6693	28.12	30.32	128.35	2.40	21.67	BDL	36.02	1.8	416.8	1.0	7.6
13	BLD	2072	20975	7147	9403	1583	9530	40.01	66.16	185.61	3.42	23.16	BDL	25.08	3.8	302.5	3.1	5.5
14	SYP	3440	21775	4859	13169	3469	19281	86.6	112.3	112.3	6.5	32.1	BDL	37.8	5.1	250.4	18.0	5.0
15	PGP	4612	25167	5232	12027	8814	24594	151.9	118.1	118.1	8.3	30.4	BDL	45.0	2.8	224.0	6.0	9.4
**Average**		**2305**	**23691**	**6179**	**12076**	**8460**	**19302**	**139.11**	**80.03**	**367.65**	**6.68**	**24.80**	**3.60**	**39.79**	**3.8**	**275.2**	**36.8**	**11.1**

**Table 2:** Low Frequency Susceptibility (IF) (10^-8^m^3^/kg), High Frequency Susceptibility (HF) (10^-8^m^3^/kg) and Frequency dependent susceptibility (FD) %.

**Location ID**	**χLF**	**χHF**	**χFD (%)**
**PKP**	58.00	56.67	2.30
**EPC**	17.67	15.50	12.26
**ARV**	815.25	810.50	0.58
**NDK**	20.75	20.17	2.81
**MTP**	31.50	30.25	3.97
**VMP**	17.33	12.83	25.96
**NVD**	9.42	7.83	16.81
**NRB**	7.75	7.42	4.30
**TZK**	9.75	6.92	29.06
**COT**	10.92	8.00	26.72
**RSP**	5.92	5.67	4.23
**STP**	7.67	7.33	4.35
**BLD**	26.92	26.42	1.86
**SYP**	6.92	6.75	2.41
**PGP**	23.17	22.00	5.04
**Average**	**71.26**	**69.62**	**9.51**

**Fig. 3:**
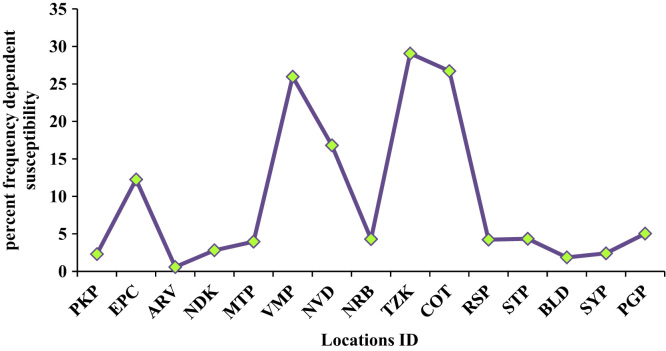
Locatons vs percent frequency dependent susceptibility.

Percent Frequency-dependent susceptibility (χ_fd_%) is a diagnostic tool to know the proportion of superparamagnetic grains in sediments [3]. Low value of χ_fd_% indicates that the magnetic properties of the samples are predominately contributed by the coarse multi-domain (MD) grains, rather than by the super-paramagnetic (SP) particles. Fig. 3 shows Location Vs percent frequency dependent susceptibility.

The correlation between the magnetic susceptibility and the heavy metal concentrations is given in Table 3. The correlation between magnetic concentration related parameters and heavy metals content reveals a causal relation between ferromagnetic oxide and heavy metals in samples. This relationship could be due to that fact that heavy metal elements are incorporated into the lattice structure of the ferromagnetic during combustion process or are adsorbed onto surface of pre-present ferrimagnetics in the environments [4].

**Table 3:** Correlation matrix between heavy metals and magnetic parameters in sediments, Coastal area, Tamilnadu.

**Variables**	**Mg**	**Al**	**K**	**Ca**	**Ti**	**Fe**	**V**	**Cr**	**Mn**	**Co**	**Ni**	**Zn**	**As**	**Cd**	**Ba**	**La**	**Pb**	**χLF**	**χFD**
**Mg**	1																		
**Al**	0.580	1																	
**K**	−0.557	−0.445	1																
**Ca**	0.781	0.552	−0.559	1															
**Ti**	0.300	0.872	−0.429	0.197	1														
**Fe**	0.587	0.933	−0.657	0.536	0.921	1													
**V**	0.340	0.886	−0.469	0.244	0.998	0.941	1												
**Cr**	0.657	0.905	−0.691	0.545	0.862	0.972	0.888	1											
**Mn**	0.393	0.898	−0.503	0.455	0.931	0.940	0.940	0.853	1										
**Co**	0.614	0.936	−0.669	0.582	0.901	0.998	0.923	0.970	0.939	1									
**Ni**	0.842	0.611	−0.736	0.854	0.320	0.641	0.368	0.727	0.439	0.667	1								
**Zn**	0.608	0.945	−0.570	0.592	0.884	0.977	0.906	0.940	0.929	0.978	0.663	1							
**As**	0.577	0.902	−0.334	0.649	0.755	0.841	0.771	0.771	0.870	0.857	0.506	0.872	1						
**Cd**	−0.049	−0.361	0.299	−0.270	−0.403	−0.434	−0.420	−0.380	−0.463	−0.439	−0.236	−0.437	−0.410	1					
**Ba**	−0.604	−0.730	0.883	−0.687	−0.610	-0.821	−0.646	−0.844	−0.706	−0.837	-0.816	−0.778	−0.614	0.364	1				
**La**	0.117	0.790	−0.340	0.035	0.970	0.830	0.959	0.761	0.878	0.803	0.179	0.791	0.650	−0.371	−0.505	1			
**Pb**	0.469	0.943	−0.487	0.518	0.920	0.959	0.935	0.906	0.964	0.961	0.527	0.961	0.905	−0.516	−0.739	0.837	1		
**LF**	−0.083	0.628	−0.130	-0.237	0.888	0.657	0.864	0.600	0.716	0.617	−0.043	0.618	0.442	−0.250	−0.295	0.945	0.683	1	
**FD**	0.096	0.005	−0.347	0.393	−0.104	0.039	−0.100	−0.037	0.073	0.070	0.283	0.046	0.087	−0.115	−0.344	−0.119	0.018	−0.264	1

*Note:* Bold values indicates significant correlation between variables

## 2 Experimental design, materials and methods

### 2.1 Study area description

**Fig. 4:**
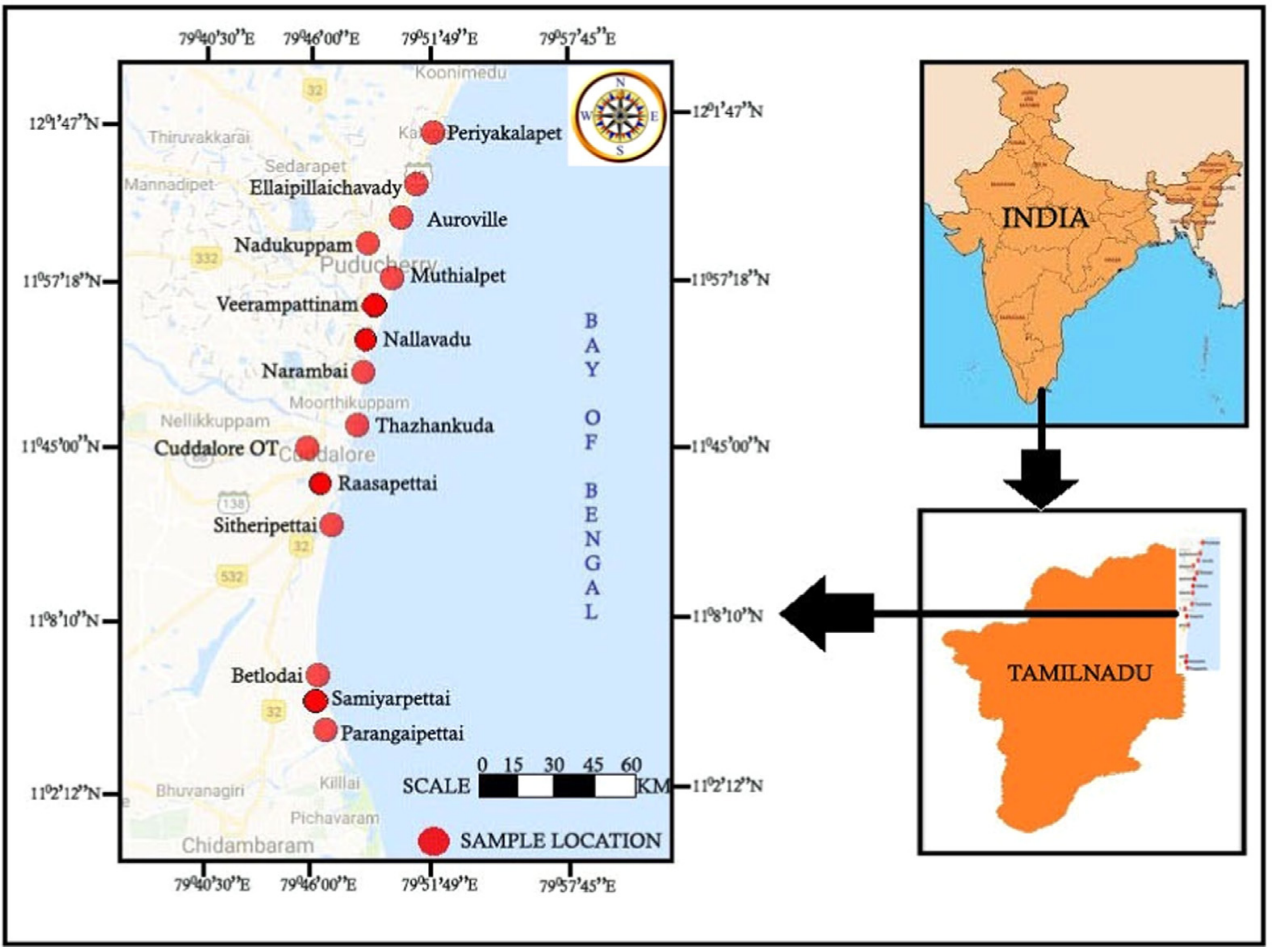
Location map of the Study Area.

The present study area covers from Periyakalapet (N:12° 1' 46.6320'' E:79° 51' 49.0032'') to Parangaipettai (N:11° 30' 0.0000'' E:79° 46' 0.0012''), East coast of Tamil Nadu. The location map of the study area is given in Fig. 4. The area between Periyakalapet and Parangipettai are represented by both depositional and erosional nature like deltaic alluvial plains, cheniers, paleo lagoonal plains and strandlines, coastal sand dunes, beaches, beach cliffs, paleo-barrier, paleotidal flats and mud flats, river mouth bars, abandoned river channels and natural levees. The beach and strandlines plain border in the west by Canaries with intervening Paleo-lagoonal plain. The principal rivers in the study area are the River Gadilam and Uppanar.

### 2.2 Sample collections

Sediment samples are collected from 15 locations along Periyakalapet to Parangaipettai Coast of Tamilnadu using a Peterson grab sampler. The grab sampler collects 10 cm thick bottom sediment layer from the seabed along the 15 locations during the pre monsoon period. Table 4 shows the geographic coordinates of the sampling locations. Garmin oregon 550, hand held Global Positioning System (GPS) was utilized for identifying the sampling locations [5], [6], [7], [8]. The collected samples were immediately transferred to polythene bags and refrigerated at 4 °C until analysis. The samples are taken to the laboratory, dried at room temperature and sieved to remove large fractions with a 1×1 mm nylon sieve [9], [10]. Then samples were oven dried at 105 °C for 2 h to a constant weight and sieved through 63 μm sieve since heavy metals are most often associated with small grains [8], [11]. The samples were then grinded to a fine powder using an agate martor. All powder samples were stored in a desiccator until they were analyzed.

**Table 4:** Latitude and longitude value of study area.

**S. No**	**Location ID**	**Name of the Location**	**Latitude (N)**	**Longitude (E)**
1	PKP	Periyakalapet	12° 1' 46.6320''	79° 51' 49.0032''
2	EPC	Ellaipillaichavady	11° 55' 54.0228''	79° 48' 19.1268''
3	ARV	Auroville	11°59'2.8422"	79°50'55.5334"
4	NDK	Nadukuppam	11°58'1.7401"	79°38'35.5103"
5	MTP	Muthialpet	11° 57' 18.2556''	79° 50' 4.1712''
6	VMP	Veerampattinam	11° 54' 5.6160''	79° 49' 36.7428''
7	NVD	Nallavadu	11° 51' 27.6014''	79°34'27.46"
8	NRB	Narambai	11° 49' 3.2520''	79° 48' 0.9216''
9	TZK	Thazhankuda	11°46'14.2020"	79°47'40.5605"
10	COT	Cuddalore OT	11° 45' 0.0000''	79° 45' 0.0000''
11	RSP	Raasapettai	11° 40' 56.2692''	79° 46' 17.5008''
12	STP	Sitheripettai	10° 30' 31.6944''	77° 13' 17.7600''
13	BLD	Betlodai	11° 21' 45.2300''	79° 32' 21.8544''
14	SYP	Samiyarpettai	11° 32' 57.2100''	79° 45' 31.8744''
15	PGP	Parangaipettai	11° 30' 0.0000''	79° 46' 0.0012''

### 2.3 Magnetic susceptibility (χ) measurements

The samples were air dried at room temperature to reduce the mass contribution of water and to avoid any chemical reactions. They were then sieved using a 1 mm sieve mesh to remove particles such as glass, plant debris, refuse and small stones [12]. The sieved samples were stored in a plastic container for further laboratory measurements. The magnetic susceptibility measurements were then carried out on the sieved samples packaged in a 10 ml plastic container at laboratory temperature. Measurements of magnetic susceptibility were made at both low (0.465 kHz) and high (4.65 kHz) frequencies using MS2B dual frequency susceptibility meter linked to a computer operated using a Multisus 2 software. All measurements were conducted in the 1.0 sensitivity settings. Each sample was measured five times in two different frequencies (low and high) and an average is calculated. For natural samples which generally exhibit a continuous and nearly constant grain size distribution, can be used as a proxy for relative changes in concentration in pedogenic fined – grained magnetic particles [13]. Hence percent-frequency dependent susceptibility (χ_fd_%) was calculated from the expression [14].

(1) $$\chi_{\mathrm{fd}}\left(\%\right)=\left[\frac{\left(\chi_{\mathrm{lf}}-\chi_{\mathrm{hf}}\right)}{\chi_{\mathrm{lf}}}\right]\times 100$$
