# Supplementary material for: Data on heavy metal and magnetic relationships in coastal sediments from South East Coast of Tamilnadu, India
Source: Data Brief. 2017 Nov 20;16:392–400. doi: 10.1016/j.dib.2017.11.056 (PMC5723266; doi:10.1016/j.dib.2017.11.056)
Supplement: Supplementary file 1 — Supplementary material [file mmc1.docx]

From

**Dr.R.Ravisankar**

Assistant Professor

Department of Physics

Government Arts College

Tiruvanamalai

E-Mail: [ravisankarphysics@gmail.com](mailto:ravisankarphysics@gmail.com)

To

**The Editor**

Data in Brief

Respected Sir

Sub: **Conﬂict of interest – Reg.**

The paper entitled “**Data on Heavy metal and magnetic relationships in coastal sediments from South East Coast of Tamilnadu, India”** is submitted for revision. I strongly declare that no conﬂict of interest associated with this manuscript.

Thank You

**Yours**

**(R.Raviankar)**
